# Effects of Adding *Bacillus coagulans* BCH0 to the Diet on Growth Performance, Tissue Structures, and Gut Microbiota in Broilers

**DOI:** 10.3390/ani15091243

**Published:** 2025-04-28

**Authors:** Zhili Niu, Linbao Ji, Yucheng Zhang, Zeyi Chen, Jiakun Shen, Zhaoyue Men, Chenlong Zhou, Peng Tan, Xi Ma

**Affiliations:** 1 College of Animal Science and Technology, China Agricultural University, Beijing 100193, China; 15776561029@163.com (Z.N.); jilinbao0126@126.com (L.J.); zhangyucheng@cau.edu.cn (Y.Z.); shenjiakun2008@126.com (J.S.); menzhaoyue@163.com (Z.M.); s20223040761@cau.edu.cn (C.Z.); tanpeng1995@cau.edu.cn (P.T.); 2 College of Life Sciences, Henan Agricultural University, Zhengzhou 450002, China; 15225180770@163.com

**Keywords:** *Bacillus coagulans*, broiler, growth performance, gut microbiota, intestinal health

## Abstract

Studies demonstrated that *Bacillus coagulans* (*B. coagulans*) as a dietary additive enhanced broiler growth performance, yet its mechanisms of action modulation remained unclear. Therefore, this study investigated effects of dietary *B. coagulans* BCH0 (1 × 10^9^ CFU/kg) on growth performance, intestinal morphology, gut microbiota, and ileal transcriptomics in Arbor Acres broilers using a completely randomized design. A total of 200 one-day-old broilers were allocated to control (Con, basal diet) and experimental (BCH0, basal diet + 1 × 10^9^ CFU/kg *B. coagulans* BCH0) groups (10 replicates/group, 10 birds/replicate) over a 42-day trial. The results revealed that BCH0 significantly increased body weights (BW) at 21 and 42 days (*p* < 0.05), improved the average daily gain (ADG) during the starter (1–21 days) and overall phases (1–42 days), and reduced the ratio of feed intake to body weight gain (F/G) across all phases (*p* < 0.05). Duodenal morphology analysis indicated a BCH0 elevated villus height (+16.9%, *p* < 0.01) and villus height/crypt depth (V/C) (*p* < 0.01) and no significant differences in crypt depth (*p* = 0.46). In the ileum, the BCH0 group exhibited a significantly greater villus height (*p* < 0.01), crypt depth (*p* < 0.05), and V/C (*p* < 0.05) than the Con group. Microbiota analysis revealed no significant differences in α-diversity or β-diversity, but phylum-level shifts involved an increase in *Firmicutes* and a reduction in *Actinobacteriota* in the BCH0 group. At the genus level, dominance shifted from *Romboutsia* (Control group) to *Lactobacillus* (BCH0 group), accompanied by marked reductions in *Turicibacter*, *Ldatus_arthromitus*, and *Rothia*. Ileal transcriptomics identified 605 differentially expressed genes, with KEGG enrichment highlighting activated nutrient assimilation pathways (*p* < 0.05), including carbohydrate, mineral, fat, and protein digestion/absorption. These findings collectively demonstrated that *B. coagulans* BCH0 enhanced broiler growth through the synergistic modulation of beneficial microbiota, the upregulation of nutrient metabolism genes, and intestinal architectural optimization, supporting its role as a sustainable microbial additive for enhancing poultry productivity and gut health.

## Simple Summary

The emergence of antimicrobial resistance and drug residues caused by antibiotic overuse has become a critical constraint on the sustainable development of animal husbandry. Probiotics have attracted significant attention due to their potential to enhance gut health and improve growth performance in livestock. However, their efficacy is highly dependent on strain specificity, dosage, and origin. Consequently, screening and validating safe and high-efficiency functional probiotics as antibiotic substitutes to optimize the feed conversion ratio (FCR) in broilers remains a key focus in animal nutrition research. In this study, *B. coagulans* BCH0 (*B. coagulans* BCH0, 1 × 10^9^ CFU/kg) was added to broiler diets to evaluate its potential as an antibiotic replacement. The results demonstrated that *B. coagulans* BCH0 significantly improved growth performance (*p* < 0.05) by modulating intestinal morphology, optimizing gut microbiota composition, and upregulating the transcription of nutrient digestion- and absorption-related genes. These findings underscored the substantial potential of *B. coagulans* BCH0 as a sustainable, antibiotic-alternative feed additive.

## 1. Introduction

Numerous studies have demonstrated that probiotics intake effectively maintains gut health [1,2]. As fundamental bioactive components, these microorganisms are strategically added to functional food matrices, including fermented dairy products and dietary additives [3]. The intake of food products enriched with probiotics can enhance intestinal immunity and improve lactose tolerance [4]. Probiotics also promote intestinal digestion and nutrient absorption, inhibit the colonization of pathogenic bacteria, prevent gastroenteritis, and improve gut microbiota [5,6,7]. Studies indicated that a daily intake of viable probiotics could modulate gut microbiota balance [8]. Studies have shown that a daily intake of at least 1 × 10^9^ CFU/kg of probiotics was effective in improving intestinal health [9].

*B. coagulans*, a significant edible probiotic, is a gram-positive, parthenogenetic anaerobic bacterium with non-pathogenic endospore-forming characteristics [10]. *B. coagulans* is highly resistant to environmental stresses and exhibits strong tolerance to high temperatures, gastric acid, and bile salts [11]. It is also widely recognized for its probiotic properties [12,13]. In addition, *B. coagulans* is not resistant to antibiotics and can be used as an substitute to antibiotics in medicine and animal husbandry [14,15]. *B. coagulans* improves digestibility by secreting enzymes such as protease, α-amylase, xylanase, and lipase, as well as producing amino acids and vitamins [16,17]. *B. coagulans* can effectively improve gastrointestinal discomfort in humans [18,19] and attenuate intestinal mucosal damage in immunosuppressed mice [20] by modulating the intestinal barrier, inflammatory response, and intestinal microbiota. Studies have demonstrated its capacity to increase the height of ileocecal villus while reducing crypt depth in weaned piglets, thereby improving nutrient absorption [21]. Adding *B. coagulans* to the feed can positively influence improvements in feed consumption and weight gain in heat-stressed broiler chickens and enhance the development effect in broiler chickens after infection with *Clostridium perfringens* and *Salmonella* [22].Compared to traditional *B. coagulans*, *B. coagulans* BCH0 exhibits high-temperature resistance, acid–bile salt tolerance, and other characteristics. Moreover, its solid-state fermentation process reduces production costs while achieving high yields. Additionally, it poses no risk of antibiotic resistance and possesses unique advantages due to its special acid-producing metabolic pathway. Although previous studies have shown that *B. coagulans* could enhance intestinal health and growth performance in broilers, the cascade mechanisms underlying this repair process remain unclear. Therefore, this study explored effects of adding *B. coagulans* BCH0 to diet on growth performance, tissue structures, and gut microbiota in broilers, which aims to investigate the safety and mechanism of *B. coagulans* BCH0.

## 2. Materials and Methods

### 2.1. Preparation of B. coagulans BCH0 Additive

The additive used in this study was a dried preparation of the *B. coagulans* BCH0 strain produced via solid-state fermentation and provided by the College of Animal Science and Technology, China Agricultural University (Beijing, China). The product contains *B. coagulans* spores and solid-state fermentation substrates, with a viable bacterial concentration of 2.52 × 10^11^ CFU/kg. The mixture was homogenized by pulverization through a 20-mesh sieve. When added to broiler feed at a 0.5% inclusion rate, this additive amount ensured a final microbial concentration of 1 × 10^9^ CFU/kg. This inclusion level (1 × 10^9^ CFU/kg) was based on evidence that *B. coagulans* enhanced the intestinal mucosal barrier function and immune responses in broilers [23,24].

### 2.2. Animals and Experimental Location

Animals were housed in metal cages (1.8 mL × 0.8 mW × 0.5 mH), each equipped with automated water-line systems (replacing conventional drinkers), troughs, and natural ventilation. The lighting regime was artificially controlled to provide 16 h of light daily, with 8 h of darkness at night. The house temperature was maintained at 33–35 °C during days 1–7 post-hatching, then gradually decreased by 2–3 °C weekly until reaching an ambient temperature (20–25 °C), which was sustained thereafter. Temperature adjustments were made based on the behavioral responses of the broilers. Relative humidity was regulated as follows: 60–70% during days 1–7, progressively reduced to 50–60% during days 8–21, and stabilized at 45–55% during days 22–42. All the broilers were housed in a single-tier cage system to ensure uniform environmental conditions across experimental groups. The broilers had *ad libitum* feed access, with routine vaccination and daily sanitation protocols maintained throughout the trial.

### 2.3. Animals and Trial Design

This experiment was managed and designed according to the Chinese Guidelines for Animal Welfare upon approval by the Institutional Animal Care and Application Ethics Committee of China Agricultural University (Approval Number: AW72205202-1-02).

This research was carried out at the experimental farm of the College of Animal Science and Technology, China Agricultural University. A completely randomized design was adopted, with a total of 200 one-day-old Arbor Acres (AA) broilers allotted to two groups, each containing 10 replicates of 10 broilers. The broilers in the control group were fed a basal diet (Con group), while the experimental group received diets containing 1 × 10^9^ CFU/kg of *B. coagulans* BCH0 through a 0.5% product inclusion rate (BCH0 group). The experiment lasted for 42 days, divided into two phases (1–21 and 22–42 days). The trial employed a corn–soybean meal-based diet, with the basal diet composition and nutritional profile detailed in Table 1.

### 2.4. Sample Collection and Assay

BW were recorded per replicate at days 1, 21, and 42. Feed intake was quantified for three phases: days 1–21, days 22–42, and days 1–42. The key metrics were calculated as follows:

Average daily gain (ADG) = (Final body weight − Initial body weight) (g)/Duration (day)

The ratio of feed intake to body weight gain (F/G) = Total feed intake (g)/Total body weight gain (g).

The broilers were fasted for 12 h with free access to water before slaughter, placed in a 40 L transparent polycarbonate chamber, and exposed to high-purity CO_2_ (>99%) through a flow meter and pressure regulator. After loss of consciousness, carotid artery cannulation was performed for exsanguination, followed by the aseptic collection of intestinal samples. Through midline celiotomy, 2 cm duodenal segments (immediately distal to the pylorus) and ileal segments (proximal to the ileocecal junction) were excised with sterile iris scissors. Tissues were immersion-fixed in 4% neutral-buffered formalin (NBF, pH 7.2) for 24–48 h at 4 °C before paraffin embedding.

Fixed tissues were dehydrated with gradient ethanol and xylene, and then paraffin was embedded. The embedded blocks were sectioned into 4 µm sections using a pathology microtome (Leica, Wetzlar, Germany). The sections were dewaxed, rehydrated using xylene and graded alcohol concentrations, and stained with hematoxylin and eosin solution (Solarbio, Beijing, China).

Tissue morphology, villus height, and crypt depth were obtained by optical microscope (Olympus BX63, Tokyo, Japan). Three sections were selected from each intestinal segment. For each tissue section, 10 morphologically intact villi and their associated crypts were randomly chosen.

Ileal chyme and mucosal specimens were aseptically collected into 2 mL cryovials, immediately flash-frozen in liquid nitrogen during transport, and archived at −80 °C. For multi-omics profiling, 1 g aliquots of ileal chyme and mucosa were submitted to Majorbio Bio-pharm Technology Co., Ltd. (Shanghai, China) for parallel 16S rRNA amplicon sequencing and transcriptomic analysis.

### 2.5. Statistical Analysis

The data on growth performance (BW, ADG, F/G) and intestinal morphological structure (villus heigh, crypt depth, V/C) were processed using SPSS 26.0 statistical software (IBM Corporation, Chicago, IL USA) for Windows, with the independent samples t-test for analysis. All data are presented as mean values, with statistical significance set at *p* < 0.05.

The 16S rRNA gene sequencing was performed using the MajorBio Cloud Platform https://cloud.majorbio.com (accessed on 26 February 2025). The alpha diversity of the gut microbiota was evaluated using indices including ACE, Chao1, Simpson, and Shannon. The beta diversity of the microbial communities was assessed via principal coordinate analysis. The relative abundances of microbial taxa at the genus level were calculated to characterize the compositional features of the gut microbiota. Differential microbial communities were further identified through LEfSe analysis. Raw transcriptomic sequencing data were analyzed for differentially expressed genes (DEGs) on the MajorBio Cloud Platform, followed by gene ontology (GO) functional annotation and KEGG pathway enrichment analysis.

## 3. Results

### 3.1. Growth Performance

As presented in Table 2, adding *B. coagulans* BCH0 to the diet significantly enhanced growth performance in AA broilers. Specifically, the BCH0 group exhibited significantly higher BW than the Con group at both 21 and 42 days of age (*p* < 0.05). Notably, the ADG was markedly increased in the *B. coagulans*-added group during the 1–21 day and 1–42 day periods (*p* < 0.05). Furthermore, the F/G demonstrated significant improvements in the treatment group across all measured intervals (1–21 days and 1–42 days) compared to the Con group (*p* < 0.05).

### 3.2. Intestinal Morphology

**Figure 1:**
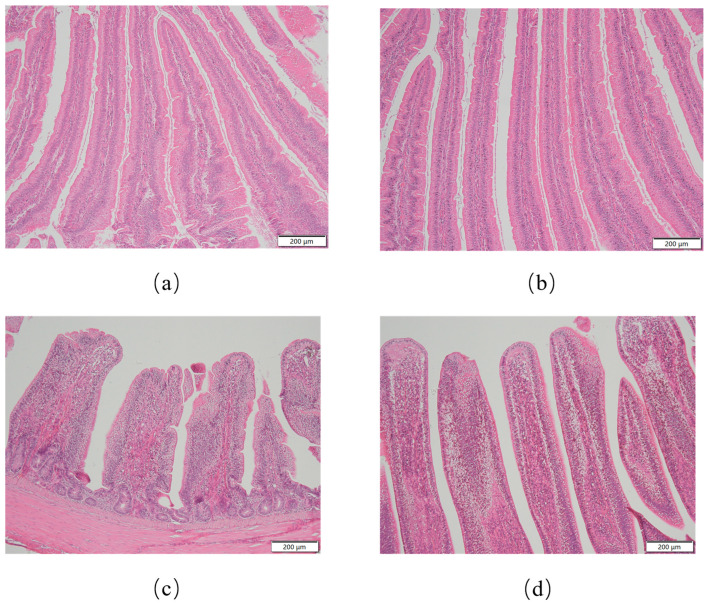
Histological morphology of intestinal villi in broilers. (**a**) Duodenal villus architecture in the control group (Con); (**b**) Duodenal villus in the *B. coagulans* BCH0-added group; (**c**) Ileal villus morphology in the Con group; (**d**) Ileal villus in the *B. coagulans* BCH0-added group. Scale bar: 200 μm.

As illustrated in Figure 1, histomorphology features of the intestinal villi in broiler chickens are presented. As presented in Table 3, duodenal morphological analysis demonstrated that the BCH0 group exhibited significantly greater villus height than the Con group (*p* < 0.01), while no significant difference in crypt depth was observed between the groups (*p* = 0.4641). Notably, the ratio of V/C was markedly elevated in the BCH0-treated animals (*p* < 0.01), indicating enhanced intestinal absorptive capacity. In the ileum, the Con group had a greater villus height (*p* < 0.01), crypt depth (*p* < 0.05), and V/C (*p* < 0.05) than the experimental group.

### 3.3. Gut Microbiota

**Figure 2:**
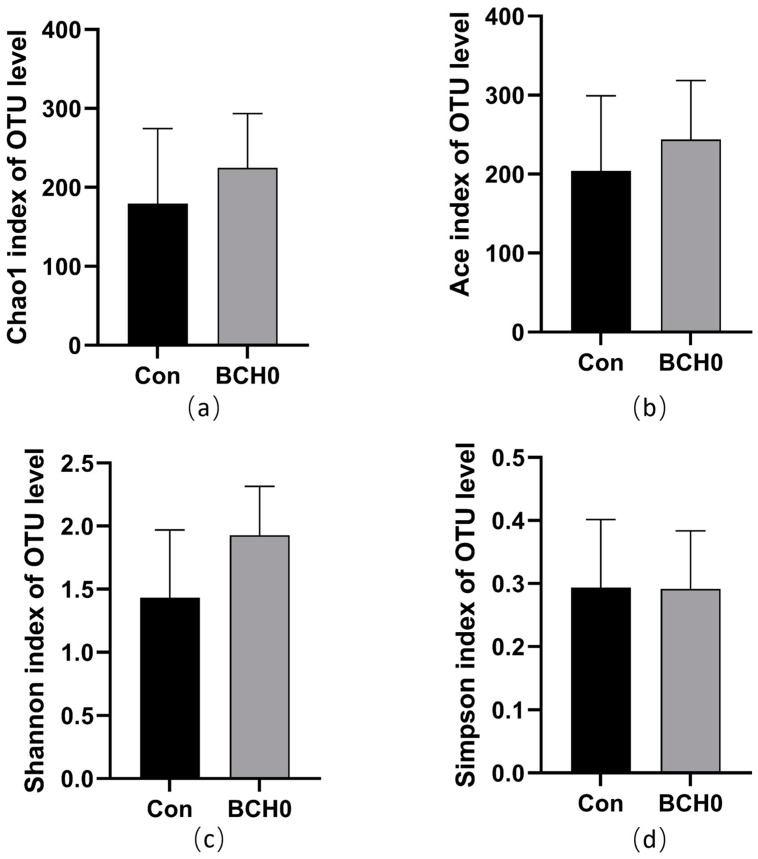
Comparative analysis of α-diversity indices between experimental groups. (**a**) Chao1 index at OTU level; (**b**) Ace index at OTU level; (**c**) Shannon index at OTU level; (**d**) Simpson index at OTU level.

As presented in Figure 2, the experimental group showed no significant differences in Chao1, Shannon, Simpson, and Ace indices compared to the control group, indicating no significant differences in α diversity (*p* > 0.05).

**Figure 3:**
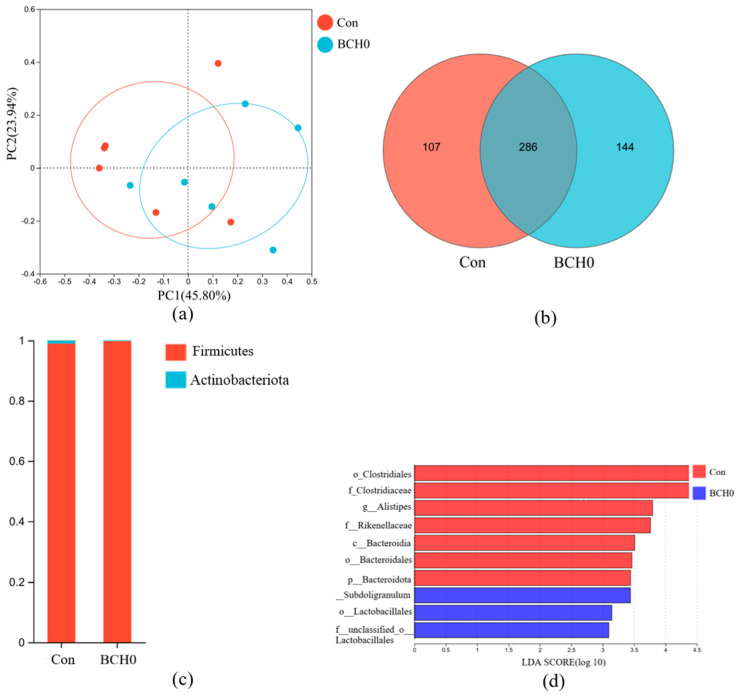
Microbial diversity analysis. (**a**) The analysis of β-diversity contrasts was made by PCoA (R^2^ = 0.1904, *p* = 0.067); (**b**) Venn diagram of OTU; (**c**) phylum-level abundance; (**d**) genus-level heatmap with dendrogram.

Comparative analysis of the beta-diversity indices between the experimental groups (Figure 3a) revealed no statistically significant differences (*p* > 0.05). Beta-diversity analysis of ileal bacterial communities, as demonstrated by principal coordinate analysis (PCoA) (R^2^ = 0.1904, *p* = 0.067), revealed no significant difference in composition between the experimental and Con groups. The proximity of data points in analyses indicated a high degree of similarity in the microbial community structure, suggesting minimal intervention-driven alterations in ileal bacterial beta diversity. At the phylum level, Firmicutes remained the dominant and most stable taxon in both the Con and experimental groups. The relative abundance of Firmicutes in the experimental group showed a marginal increase, while Actinobacteriota displayed a slight reduction. Hierarchical clustering analysis of the dominant microbiota (Figure 3c) revealed partial segregation between the Con and *B. coagulans*-added groups, with subsets of samples clustered by treatment. This pattern suggested moderate intra-group homogeneity in genus-level microbial composition, although overlapping clusters highlight shared taxonomic features between groups. Bar plot visualization further demonstrated genus-specific shifts-*Romboutsia* was predominant in the Con group, whereas *Lactobacillus* emerged as the dominant genus in the experimental group.

### 3.4. Intestinal Tissue Gene Transcription

**Figure 4:**
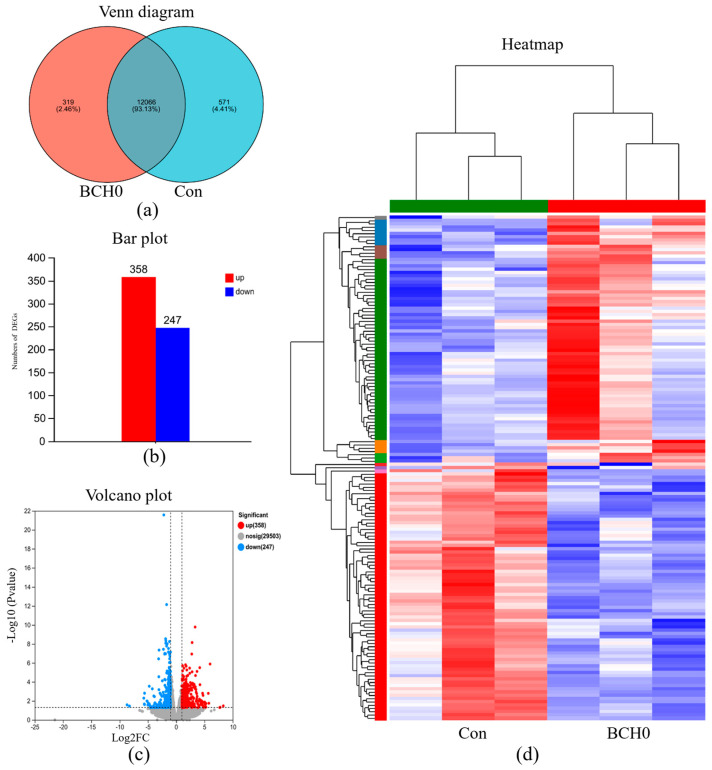
Transcriptome analysis of the *B. coagulans* BCH0 group. (**a**) Venn diagram: overlap of differentially expressed genes (DEGs); (**b**) Bar plot: quantitative comparison of regulated differentially expressed genes (DEGs); (**c**) Volcano plot: visualization of DEG significance versus magnitude. (**d**) Heatmap: hierarchical clustering of DEG expression profiles.

As shown in Figure 4, in the transcriptional analysis of ileum tissues, the Venn diagram (Figure 4a) reveals that 12,066 genes (93.13%) were commonly expressed in both the Con and BCH0 groups, while 319 unique genes (2.46%) are specific to the BCH0 group and 571 genes (4.41%) are exclusive to the Con group. Furthermore, the differential expression bar chart (Figure 4b) and volcano plot (Figure 4c) demonstrate 358 significantly upregulated genes and 247 downregulated genes in the BCH0 group compared to the Con group. This distinct imbalance toward upregulated genes suggested that BCH0 treatment predominantly enhanced transcriptional activity in ileum tissues.

**Figure 5:**
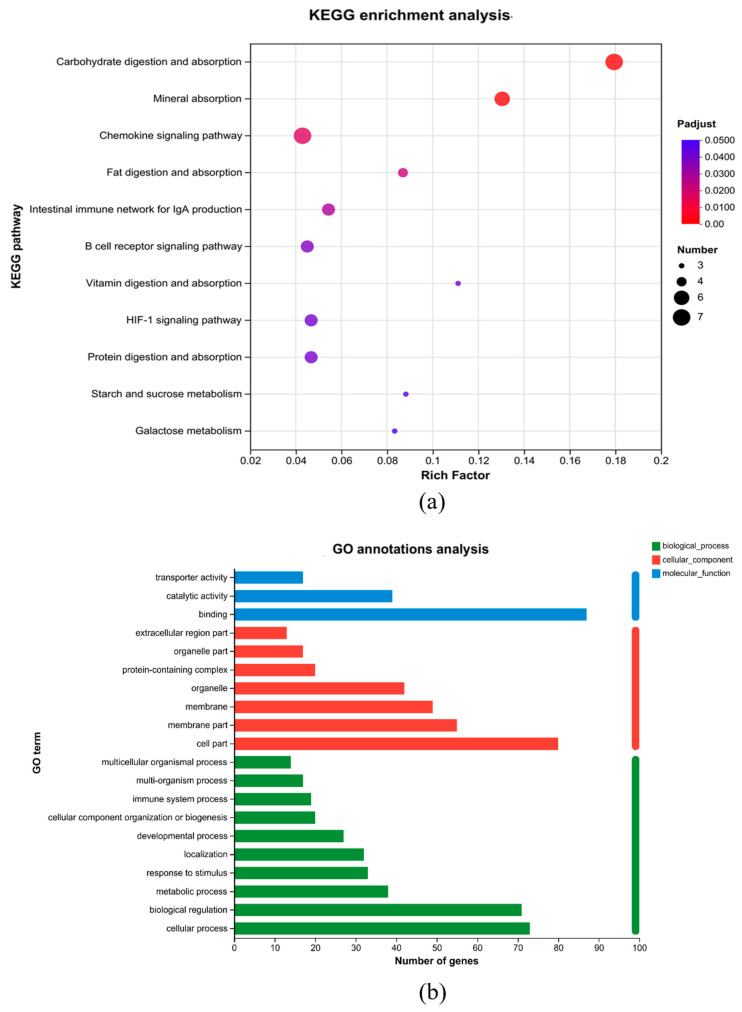
Transcriptome analysis of the *B. coagulans* BCH0 group. (**a**) Gene ontology (GO) term enrichment analysis of the DEGs. (**b**) KEGG enrichment analysis. The criteria for filtering KEGG pathways with diversities is *p* < 0.05.

KEGG and GO enrichment analyses are presented in Figure 5a and Figure 5b, respectively. GO enrichment analysis on the differentially expressed genes was performed and shows that the enrichment of differentially expressed genes in biological processes (BP) mostly includes the cellular process, biological regulation, and the metabolic process. To further examine the role of differentially expressed genes, KEGG enrichment analysis was performed. It displayed the top 11 differentially expressed pathways. These included carbohydrate digestion and absorption, mineral absorption, fat digestion and absorption, an intestinal immune network for IgA generation, a B-cell receptor signaling pathway, vitamin digestion and absorption, protein digestion and absorption, starch and sucrose metabolism, and galactose metabolism.

## 4. Discussion

Experimental evidence and meta-analytical reviews consistently demonstrated that probiotics represent a viable antibiotic-alternative strategy for enhancing growth performance in livestock [25,26]. The current investigation revealed that adding *B. coagulans* BCH0 to the diet significantly improved key growth parameters in AA broilers, as evidenced by an increased final BW, a higher ADG, and an optimized feed conversion ratio [27,28]. It is noteworthy that the substantial ADG improvements during both the starter phase (1–21 days) and the entire trial period (1–42 days), indicating probiotic efficacy during early developmental stages. These findings align with previous reports documenting a 4.6–5.3% ADG increase when *B. coagulans* was added to broiler diets [29,30]. The BCH0 group maintained consistent F/G improvements across all growth phases, underscoring this strain’s capacity to enhance nutrient utilization efficiency throughout the production cycle. The mechanistic basis for these improvements appears multifactorial. *B. coagulans* exhibits exceptional gastrointestinal survival capabilities due to its acid–bile resistance, facilitating intestinal colonization and the subsequent production of bioactive metabolites. A study demonstrated that intestinal colonization by *B. coagulans* stimulates endogenous lipase and protease secretion, enhancing the hydrolysis of complex dietary substrates [31]. From an applied perspective, the demonstrated growth enhancement carries dual implications: economic benefits through improved feed efficiency and environmental advantages via a reduced reliance on growth-promoting antibiotics. These findings substantiate *B. coagulans* BCH0 as a sustainable growth promoter in modern poultry production systems.

The small intestine serves as the primary site for nutrient absorption, with its functional efficiency being directly influenced by intestinal structural integrity. The development of intestinal villi plays a decisive role in determining digestive capacity. Morphological parameters such as villus height and crypt depth in the intestinal mucosa represent the most direct indicators for evaluating intestinal morphology [32]. A well-developed villus architecture characterized by intact, elongated villi and appropriate crypt depth facilitates optimal nutrient assimilation. Conversely, epithelial cell damage in the intestinal mucosa leads to significant villus shortening and crypt depth enlargement. This morphological alteration reflects the compensatory proliferation of crypt stem cells, which actively regenerate to repair the damaged villus epithelium [33,34]. Previous studies have found that dietary *B. coagulans* contributed to the preservation of normal intestinal morphology by antagonizing pathogenic bacterial colonization and counteracting toxin invasion [35]. Morphological analysis revealed that adding *B. coagulans* BCH0 to the diet significantly improved the intestinal architecture compared to the control group. In the duodenum, the BCH0 group demonstrated a markedly increased villus height (*p* < 0.01) and a reduced crypt depth, resulting in the elevated V/C. This enhancement was particularly pronounced in the ileum, where both the villus height and V/C showed a significant increase. These structural improvements align with established probiotic mechanisms, as prior studies confirm that adding *B. coagulans* BCH0 to the diet promotes villus elongation while reducing crypt depth, ultimately optimizing mucosal morphology for enhanced nutrient absorption [36,37,38]. The observed intestinal improvements might be attributed to the unique sporulation capacity of *B. coagulans*, enabling effective intestinal colonization. Upon reaching the gastrointestinal tract, BCH0 modulated microbial homeostasis through the biosynthesis of antimicrobial compounds, digestive enzymes, and bioactive metabolites. This multipronged action enhances luminal nutrient bioavailability, mitigates epithelial damage via anti-inflammatory mediators, and stimulates trophic factors for villus development [39,40]. Collectively, our findings substantiated that the dietary addition of *B. coagulans* BCH0 effectively preserved the intestinal mucosal architecture by synergistically reinforcing digestive competence, barrier function, and epithelial regeneration capacity.

Studies have demonstrated that gut microbiota played a pivotal role in nutrient digestion and absorption, the regulation of host fat deposition, the promotion of intestinal epithelial cell renewal, and immune system enhancement [20,41,42]. Probiotic addition has been shown to influence gut flora characteristics, particularly in microbial diversity, community composition, and relative abundance, with dietary probiotics serving as an effective modulator of microbial diversity in animals [43]. Our analysis of ileal microbiota revealed subtle but meaningful compositional changes following the addition of *B. coagulans* BCH0 to the diet. The experimental group showed no significant differences in the Chao1, Shannon, Simpson, and Ace indices compared to the control (Con) group, indicating no significant differences in α diversity (*p* > 0.05). The beta-diversity analysis via principal coordinate analysis (PCoA, R^2^ = 0.1904, *p* = 0.067) demonstrated no statistically significant differences in microbial composition between the experimental and control groups. At the phylum level, Firmicutes maintained dominance in both groups, while genus-level analysis revealed a notable ecological shift, with *Lactobacillus* replacing *Romboutsia* as the predominant genus in added subjects. This selective enrichment of *Lactobacillus*, a genus well-documented for its beneficial effects on intestinal health regulation, immune enhancement, and growth promotion in broiler chickens, suggests that *B. coagulans* BCH0 exerts targeted modulatory effects on ileal microbial communities [44]. The mechanism might be that *B. coagulans* enhances the growth of *Lactobacillus* by consuming oxygen to create an anaerobic environment while producing lactic acid and short-chain fatty acids that modulate the gut pH and supply metabolic substrates, collectively supporting the colonization of lactobacilli [45,46]. The efficacy of *B. coagulans* BCH0 may vary between cage and litter rearing systems due to differences in microbial competition and colonization resistance [47,48]. In cage systems, where gut microbiota diversity is typically lower, *B. coagulans* BCH0 may establish more effectively, potentially exerting stronger probiotic effects on growth performance and immune modulation. Conversely, in litter systems, the richer microbial environment could enhance competition, limiting *B. coagulans* BCH0 colonization but possibly synergizing with native Actinobacteria. Thus, *B. coagulans* BCH0 may require higher doses or complementary strategies in litter systems to overcome microbial interference, whereas cage-reared birds might benefit more from direct probiotic addition [49]. The observed microbial modifications align with established probiotic mechanisms, including pathogen inhibition, the structural optimization of gut microbial communities, and subsequent improvements in intestinal health parameters [50]. These findings collectively indicate that adding *B. coagulans* BCH0 to the diet induces specific, genus-level microbial adaptations while maintaining the overall community architecture, demonstrating the potential for the precision modulation of gut microbiota through probiotic interventions.

Through analysis of the transcriptome of the ileal tissues of AA broiler chickens, the differential pathways indicated that *B. coagulans* BCH0 improved the absorption and utilization of nutrients in AA broilers, promoted the improvement of broiler production performance, and had an impact on the intestinal immune function of AA broilers. These benefits included disrupting latent pathogens, enhancing barrier function and producing neurotransmitters [51]. We analyzed the transcriptome data by KEGG enrichment. After analysis, we found that carbohydrate digestion and absorption, mineral absorption, fat digestion and absorption, the intestinal immune system for IgA generation, the B-cell receptor signaling pathway, vitamin digestion and absorption, protein digestion and absorption, starch and sucrose metabolism, and galactose metabolism were upregulated. Previous studies have demonstrated that adding *B. coagulans* BCH0 to the diet alleviated inflammation, inhibited tumorigenesis, and enhanced immune function [52].

## 5. Conclusions

In this study, we demonstrated that the dietary addition of *B. coagulans* BCH0 activated the transcriptional expression of nutrient digestion-related genes, restored the intestinal morphology and V/C, and enhanced the colonization of beneficial microbial taxa such as *Lactobacillus*. These synergistic effects collectively increased the ADG of broilers and optimized the feed conversion ratio, highlighting its potential as a sustainable microbial additive for enhancing poultry productivity and gut health.

## Figures and Tables

**Table 1:** The ingredients and nutritional components of the diet (DM basis).

Ingredients	Content	
	0–21 d	22–42 d
Corn	58.04	64.06
Soybean meal	32.00	26.43
Fish meal	2.00	2.00
Soybean oil	3.40	3.40
CaHPO_4_	1.45	1.04
Limestone	1.30	1.35
NaCl	0.30	0.30
Premix ^1^	1.00	1.00
L-lysine HCl	0.36	0.33
DL-methionine	0.15	0.09
Total	100	100
Nutritional components ^2^		
Metabolizable energy (MJ/Kg)	12.34	12.60
Crude protein (%)	20.12	18.02
Ca (%)	1.00	0.79
P (%)	0.46	0.34
Lysine (%)	1.14	0.95
Methionine (%)	0.46	0.34

^1^ The premix provided the following per kg diet: VA 10 000 IU; VD 2 000 IU; VE 20 IU; VK 1 mg; VB_12_ 0.01 mg; Fe 100 mg; Cu 10 mg; Mn 120 mg; Zn 100 mg; I 0.7 mg; Se 0.3 mg; thiamine 2 mg; riboflavin 8 mg; pantothenic acid 10 mg; nicotinic acid 35 mg; pyridoxine 3.5 mg; biotin 0.2 mg; folic acid 0.6 mg. ^2^ Nutrient levels were calculated values.

**Table 2:** Effects of *B. coagulans* BCH0 on the growth performance of AA broilers.

Items	Days	Con	BCH0	SEM	*p*-Value
BW (g)	1	46.90	46.55	0.41	0.5401
	21	857.23 ^a^	925.06 ^b^	16.20	0.0001
	42	2405.96 ^a^	2531.02 ^b^	59.01	0.0400
ADG(g/d)	1–21	38.59 ^a^	41.83 ^b^	0.78	0.0010
	1–42	56.17 ^a^	59.15 ^b^	3.40	0.0470
F/G	1–21	2.06 ^b^	1.83 ^a^	1.40	0.0480
	1–42	2.24 ^b^	1.95 ^a^	0.12	0.0214

^a, b^: Means within rows with different superscripts differ significantly (*p* < 0.05). SEM, standard error of the mean (n = 10).

**Table 3:** Effects of *B. coagulans* BCH0 on tissue structures of AA broilers.

Items	Segment	Con	BCH0	SEM	*p*-Value
Duodenum	villus height (μm)	1420.81 ^a^	1660.18 ^b^	35.25	0.0001
	crypt depth (μm)	273.99	256.91	29.92	0.4641
	V/C	5.18 ^b^	6.49 ^a^	0.19	0.0001
Ileum	villus height (μm)	750.44 ^a^	959.64 ^b^	27.85	0.0001
	crypt depth (μm)	157.27 ^a^	173.69 ^b^	5.84	0.0100
	V/C	4.81 ^a^	5.53 ^b^	0.24	0.0001

^a, b^: Means within rows with different superscripts differ significantly (*p* < 0.05). SEM, standard error of the mean (n = 10).

## Data Availability

The datasets supporting the conclusions of this article are available in the NCBI Sequence Read Archive (SRA) repository under accession number PRJNA1255734 and PRJNA1255727 (available on 26 April 2025).
